# Sports Participation and Juvenile Delinquency: A Meta-Analytic Review

**DOI:** 10.1007/s10964-015-0389-7

**Published:** 2015-11-23

**Authors:** Anouk Spruit, Eveline van Vugt, Claudia van der Put, Trudy van der Stouwe, Geert-Jan Stams

**Affiliations:** Department of Forensic Child and Youth Care Sciences, University of Amsterdam, Nieuwe Achtergracht 127, PO Box 15776, 1001 NG Amsterdam, The Netherlands

**Keywords:** Sports participation, Juvenile delinquency, Multilevel meta-analysis, Review

## Abstract

Participation in sports activities is very popular among adolescents, and is frequently encouraged among youth. Many psychosocial health benefits in youth are attributed to sports participation, but to what extent this positive influence holds for juvenile delinquency is still not clear on both the theoretical and empirical level. There is much controversy on whether sports participation should be perceived as a protective or a risk factor for the development of juvenile delinquency. A multilevel meta-analysis of 51 published and unpublished studies, with 48 independent samples containing 431 effect sizes and *N* = 132,366 adolescents, was conducted to examine the relationship between sports participation and juvenile delinquency and possible moderating factors of this association. The results showed that there is no overall significant association between sports participation and juvenile delinquency, indicating that adolescent athletes are neither more nor less delinquent than non-athletes. Some study, sample and sports characteristics significantly moderated the relationship between sports participation and juvenile delinquency. However, this moderating influence was modest. Implications for theory and practice concerning the use of sports to prevent juvenile delinquency are discussed.

## Introduction

A large number of adolescents is participating in sports activities. The 2011–2012 National Survey of Children’s Health showed that 63 % of the 12- to 17-year olds participated in sports lessons or a sports team. Generally, sports participation is perceived as a positive leisure activity that is associated with positive (psychosocial) health outcomes in adolescents (Eime et al. 2013; Janssen and LeBlanc 2010). However, the public opinion about adolescent athletes’ behavior is ambiguous. On the one hand it is believed that sports have a positive influence on the development of youth, and therefore, youth who participate in sports activities are expected to have a lower risk of engaging in delinquent behavior than youth who do not participate in sports activities (Faulkner et al. 2007; Miller et al. 2007; Shields and Bredemeier 1995). This assumption has led local governments and institutions all over the world to offer youth sports activities and interventions to prevent juvenile delinquency (Cameron and MacDougall 2000; Hartmann 2003; Kelly 2013; Miller et al. 2007; Nichols 2007; Sandford et al. 2006). On the other hand, due to negative reports in the media about athletes’ drug use and anti-social behavior, sports participation has often been linked to (juvenile) delinquency (Benedict and Klein 1997; Hughes and Shank 2005; Kwan et al. 2014; Yesalis and Bahrke 2000).

This division in views has led some researchers to test the assumptions on the association between sports participation and juvenile delinquency in order to understand if, and how sports participation is contributing to the occurrence of juvenile delinquency (Miller et al. 2007). So far, empirical evidence is inconclusive (Coakley 2002; Farb and Matjasko 2012; Gardner et al. 2009; Nichols 2007), and to date, there is no systematic review on the association between sports participation and juvenile delinquency available. It remains unclear whether sports participation is either positively or negatively associated with delinquent behavior among youth or whether no associations exist at all. Therefore, the aim of the current meta-analysis is to examine the relationship between sports participation and juvenile delinquency.

### Theoretical Framework

Sports participation and delinquency are important developmental themes in adolescence. During adolescence, youth become more autonomous from their parents and the influence of the home environment shifts towards the afterschool, peer, and leisure setting (Fredricks and Eccles 2008). At the same time, the development and incidence of delinquent behaviors peaks (Moffitt 1993). Studying the relationship between sports participation and juvenile delinquency is therefore particularly relevant during adolescence.

Over the years, scientists have developed multiple theories about the relationship between sports participation and delinquency during adolescent years. Some of these theories support the idea that sports participation is associated with less juvenile delinquency. For example, Hirschi’s (1969) social bonds theory claims that individuals with stronger bonds to society are less likely to engage in delinquency, as delinquency may put these valuable bonds at risk. Four elements in Hirschi’s (1969) theory are central: attachment, commitment, belief, and involvement. Some (Agnew and Petersen 1989; Hass 2001) argue that sports participation has a positive influence on all four elements. Sports are supposed to enhance the *attachment* to significant others as youth become members of a team, generally supervised by a coach who is closely related to all members. When youth are *committed* to conventional activities, such as sports, they may refrain from deviant acts as this may jeopardize their opportunity to participate in sports. *Beliefs* in society’s values may be strengthened by sports participation, as similar rules, norms, and values are being practiced in the sports context. Finally, *involvement* in sports is thought to protect from juvenile delinquency because athletes are simply too occupied to engage in delinquency (Hirschi 1969). Similar arguments can be found in the boredom theory (Schafer 1969) and the routine activities theory (Cohen and Felson 1979). The boredom theory states that juvenile delinquency may originate from boredom, and because athletes are just too busy to become bored, they might refrain from delinquency (Schafer 1969). The routine activities theory assumes that delinquency occurs when there are opportunities, and thus engagement in structured activities, such as sports, reduces one’s time and opportunity to engage in delinquency (Cohen and Felson 1979).

Furthermore, the “sports build character”-idea claims that sports may contribute to the development of positive traits, skills, and virtues in youth (Sage 1990; Segrave 1983). For example, Arnolds (1994) states that athletes judge what is right or wrong according to the rules of the game, care for the wellbeing of all participants in the game, and choose an appropriate moral action. By committing to the internal goals and standards of the sports, athletes practice the exercise of virtues, such as honesty and fairness (Arnold 1994). It has been mentioned as well that sports teach youth to deal with setbacks, stimulate perseverance and self-control, enhance the co-operation between peers, and increase peer acceptance (Kreager 2007; Shields and Bredemeier 1995). Furthermore, higher rates of initiative and emotional regulation have been found among young athletes compared to non-athletes (Larson et al. 2006; Shields and Bredemeier 1995). Finally, there is a widely supported assumption that sports participation will lead to more self-esteem in adolescents (Adachi and Willoughby 2014; Findlay and Bowker 2009), making them less vulnerable to negative peer influences (Wild et al. 2004). Therefore, many scholars hypothesize that sports participation can reduce juvenile delinquency (Donnellan et al. 2005). In sum, there are several theories supporting the assumption that sports participation is associated with less juvenile delinquency.

On the contrary, scholars have suggested that sports participation is related to more juvenile delinquency. It has been argued that the competitive element in the sports context can actually encourage immoral behavior. Injuring an opponent, cheating, or using illegal performance-enhancing products may be rewarding if that leads to winning a game (Boardley and Kavussanu 2011; Lee et al. 2007; Nucci and Young-Shim 2005; Shields and Bredemeier 1995). Bredemeier et al. (1986) found that children participating in contact sports showed lower levels of moral judgment. As lower levels of moral judgment have been found in juvenile delinquents (Stams et al. 2006) and criminal offense recidivism (Van Vugt et al. 2011), it can be argued that certain sports activities may enhance the risk for juvenile delinquency. Finally, the culture of some sports teams have been associated with excessive alcohol consumption (Kwan et al. 2014), increasing the likelihood of engaging in delinquent behaviors (Barnes et al. 2002). All in all, there are also theories supporting the assumption that sports participation is associated with more juvenile delinquency.

Further, there are scholars who have argued that sports participation is not associated with delinquency at all, and they have criticized the theories supporting a protective influence of sports participation on juvenile delinquency. The idea that young athletes are just too busy with sports to commit crimes (Hirschi 1969; Schafer 1969) has been rejected for being too simplistic. Tappan (1949) mentioned that “If a child is disposed towards law violation … it will require much more than games and sports to do anything effective about it” (p. 150). Furthermore, it has been questioned if young athletes are in fact too busy to commit delinquent acts (Agnew and Petersen 1989; Chapple et al. 2005; Tappan 1949), because “even highly organized recreational activities do not absorb enough of the energy or time of a child to reduce appreciably his opportunities to engage in delinquency” (Tappan 1949, p. 150). The idea that sports build character, and therefore protect against the development of juvenile delinquency, has been questioned too. One of the concerns about this theory is that the potential skills and virtues that are learned in the sports context may not be carried over to situations outside this context, and that the influence of sports might not be large enough to change behavioral patterns and personality traits (Shields and Bredemeier 1995; Tappan 1949). Therefore, sports participation and juvenile delinquency may not be related to each other at all.

Summarizing the abovementioned theories on the relationship between sports participation and juvenile delinquency, it can be concluded that from a theoretical point of view there is much contradiction regarding the association between sports participation and juvenile delinquency. Previously conducted empirical research has not shed a clear light on the relationship between sports participation and juvenile delinquency either, as empirical research has shown mixed and inconclusive results (Coakley 2002; Farb and Matjasko 2012; Miller et al. 2007). Primary studies have found that sports participation was positively (Begg et al. 1996; Fauth et al. 2007; Kelley and Sokol-Katz 2011), negatively (Buhrmann 1977; Segrave and Hastad 1982), or not associated (Barnes et al. 2007; Gardner et al. 2009; Miller et al. 2007; Wong 2005) with juvenile delinquency. To determine the role of sports participation in the occurrence of juvenile delinquency, the relationship between sports participation and juvenile delinquency should be clarified.

## Current Study

To date, no systematic review has been conducted to examine the relationship between sports participation and juvenile delinquency, although there are multiple primary studies on the relationship between sports participations and juvenile delinquency available. This meta-analytic review aims to answer the question whether there is a relationship between sports participation and juvenile delinquency by synthesizing the previously conducted studies. Further, as the results of previous studies are inconsistent (Coakley 2002; Farb and Matjasko 2012; Gardner et al. 2009), there is particular interest to assess which factors moderate the association between sports participation and juvenile delinquency. A meta-analysis can provide a summary of this previously conducted research more adequately and precisely than a narrative review (Lipsey and Wilson 2001), and it is an appropriate method to quantify and analyze inconsistencies. Therefore, we chose to conduct a meta-analysis to assess the strength of the relationship between sports participation and juvenile delinquency, and to examine factors that may moderate this association.

The current meta-analysis addressed the following research questions: (1) What is the strength and direction of the relationship between sports participation and juvenile delinquency? (2) Which offense, study, sample, and sports characteristics moderate the relationship between sports participation and juvenile delinquency?

## Method

### Inclusion Criteria

Multiple inclusion criteria were formulated to select the studies for this meta-analysis. First, juvenile delinquency has been operationalized as criminal behavior (i.e., a violation of the law) by a minor outside the sports context. We excluded other types of deviant behavior (for example, behavioral problems, status offenses, antisocial behavior, substance use, or aggression) from the current meta-analysis to increase the comparability of the outcome measures in the studies (Hofer and Piccinin 2009). Second, the study had to report about the relationship between sports participation and juvenile delinquency in a way that made it possible to calculate an effect size. We included studies reporting on adjusted statistics (the reported statistic is controlled for background characteristics) and unadjusted statistics (the reported statistic is not controlled for background characteristics). Third, the mean age of the sample had to be between age 12 and 18. Fourth, the study had to contain both athlete and non-athlete samples, and both delinquent and non-delinquent samples, or samples of the general population of adolescents. Finally, the variables of interest had to be measured on the individual level. Studies measuring sports participation combined with other types of activity participation and studies measuring the effect of a sports intervention were excluded.

### Selection of Studies and Handling Publication Bias

All studies addressing the relationship between sports participation and delinquency in juveniles which were published before October 2015 were included in the current meta-analysis. Nine electronic databases were searched by the first author: ScienceDirect, Web of Knowledge, Ovid (including ERIC), Picarta, Wiley, Google Scholar, Proquest (including Dissertations and Theses and Sociogical Abstracts), EBSCOhost (including SPORTDiscus), and Narcis. The search string included three combined variables: a sports element, a delinquency element, and an age element. For the sports element, the following keywords were used: sport*, leisure, physical activity, after-school, or extracurricular. For the delinquency element, the following keywords were used: delinquen*, aggressi*, externali*, crim*, deviant, behavioral problem, offend*, or antisocial. For the age element, the keywords youth*, juvenile, adolescen*, or child were used. In most electronic databases it was possible to search only in specific parts of the publications (i.e., in the title, abstract, or key-words). In case the database offered this search option, we selected this option to reduce the number of unsuitable hits.

A common problem in performing a meta-analysis is that studies may not have been published because of non-significant or unfavorable findings, the so called “publication or file drawer bias” (Rosenthal 1995). Therefore, it is possible that the studies included in the meta-analysis are not an adequate representation of all previous studies that have been conducted. In order to prevent the problem of publication bias, we screened unpublished studies by searching the Proquest Dissertations and Theses database. Additionally, reference sections of review studies on leisure participation and behavioral problems were searched for qualifying studies. Finally, the publication lists of some experts on sports and antisocial behavior were checked for eligible studies. In case we found unpublished studies, we emailed the authors for the full text of the study, or ordered the study from the Proquest Dissertation Express.

The first author conducted the screening and selection process. When in doubt, the last author was consulted. "Appendix" presents a flow chart of the search. The initial search resulted in 414 articles, which also contained review and qualitative studies. This was narrowed down to 181 articles by inspection of the abstract and the method section, including studies examining all kinds of deviant behavior. After excluding the studies with other types of deviant behavior than delinquent behavior, 73 articles remained for thorough investigation. Finally, a total of 51 studies (with 48 independent samples, 431 effect sizes, and 132,366 participants) met the inclusion criteria. Five studies had overlapping samples; three studies (Daigle et al. 2007; Kelley and Sokol-Katz 2011; Tolk 2003) used the same waves of the Add Health-trial, and two studies (Gardner et al. 2009; Fauth et al. 2007) both used data from the Project on Human Development in Chicago Neighborhoods. Studies with overlapping samples were given the same study number. Table 1 shows the study characteristics of the included studies.

**Table 1:** Study characteristics of included studies

Study	Study characteristics							Sample characteristics			Sports characteristics			
	Year	*N*	# *r* (*M*)	Peer review	Impact factor	Design	Outcome	Age	% male	% minority	Measure IV	Team sports	Contact sports	Setting
Agnew	1989	599	6 (−.078)	Yes	1.782	CROSS	Mix	UN	50.2	0.0	INTEN	TEAM	UN	OUT
Barnes	2007	606	2 (.020)	Yes	2.777	CROSS	OD	>16	45.2	30.0	INTEN	UN	UN	OUT
Baumert Jr	1998	6849	4 (.039)	Yes	2.748	CROSS	Mix	UN	48.6	56.8	DICHO	UN	UN	OUT
Begg	1996	527	16 (.112)	Yes	4.171	LONG	OD	>16	Mix	3.0	INTEN	Mix	UN	OUT
Booth	2008	1366	3 (−.028)	Yes	1.192	CROSS	VIOL	UN	Mix	5.8	DICHO	UN	UN	UN
Buhrmann	1977	857	7 (−.159)	Yes	–	CROSS	OD	Mix	0	–	Mix	UN	UN	SCH
Buhrmann	1978	551	9 (.185)	Yes	–	CROSS	OD	Mix	0	–	Mix	UN	UN	SCH
Caldwell	2006	475	2 (.015)	Yes	0.651	CROSS	PRO D	>16	49.0	–	INTEN	UN	UN	OUT
Carr	2001	76	1 (.083)	Yes	1.638	CROSS	OD	<16	56.6	87.0	DICHO	TEAM	CON	UN
Chapple	2005	577	10 (−.024)	Yes	1.151	CROSS	Mix	UN	Mix	13.6	INTEN	TEAM	UN	UN
Choquet	2003	5473	30 (.069)	Yes	–	CROSS	Mix	<16	Mix	–	DICHO	UN	UN	OUT
Crean	2012	2512	4 (.040)	Yes	1.373	CROSS	Mix	<16	49.4	88.0	INTEN	TEAM	UN	UN
Daigle	2007	3422	6 (.043)	Yes	1.452	CROSS	Mix	<16	Mix	28.5	INTEN	UN	UN	UN
Davis	2013	1551	12 (.021)	Yes	0.483	CROSS	Mix	UN	53.2	21.1	DICH	TEAM	Mix	SCH
Faulkner	2007	3796	2 (.060)	Yes	2.855	CROSS	OD	<16	46.9	–	INTEN	UN	UN	UN
Fauth	2007	1315	4 (.140)	Yes	3.782	LONG	OD	<16	48.9	85.4	INTEN	UN	UN	OUT
Gardner	2009	1344	8 (−.006)	Yes	3.782	LONG	Mix	UN	Mix	85.0	DICHO	TEAM	UN	UN
Gies	2003	3217	10 (−.002)	No	–	LONG	OD	UN	100	–	Mix	Mix	Mix	SCH
Hallingberg	2014	137	1 (−.409)	Yes	1.475	CROSS	OD	<16	100	–	DICHO	TEAM	UN	UN
Harrison	2003	47,434	3 (−.103)	Yes	1.659	CROSS	PRO D	UN	Mix	15.5	DICHO	TEAM	UN	SCH
Hass	2001	822	6 (.032)	No	–	CROSS	Mix	<16	53.2	21.1	INTEN	TEAM	UN	OUT
Jennings	2009	747	2 (.005)	Yes	2.378	CROSS	PRO C	>16	Mix	100	DICHO	UN	UN	UN
Junger-Tas	1990	994	1 (.000)	No	–	CROSS	PET	<16	UN	–	INTEN	UN	UN	UN
Kelley	2011	4751	11 (.059)	Yes	–	Mix	Mix	UN	UN	–	DICHO	UN	UN	SCH
Kruissink	1988	528	18 (.044)	No	–	CROSS	Mix	<16	Mix	–	DICHO	UN	UN	Mix
Kwon	2014	407	1 (.06)	Yes	2.777	CROSS	OD	<16	46.0	0.0	INTEN	IND	No	UN
Landers	1978	521	3 (−.232)	Yes	2.270	CROSS	OD	UN	UN	–	DICHO	TEAM	UN	SCH
Levin	1995	2436	45 (−.011)	Yes	1.613	CROSS	Mix	>16	Mix	27.0	DICHO	UN	Mix	SCH
Luthar	2006	164	6 (.088)	Yes	3.782	CROSS	OD	UN	Mix	7.0	INTEN	UN	UN	OUT
MacRae	2011	123	1 (−.103)	Yes	1.016	LONG	OD	>16	82.9	26.0	DICHO	UN	UN	UN
Mays	2009	13956	2 (.098)	Yes	2.748	CROSS	PET	UN	Mix	–	DICHO	TEAM	UN	UN
Meenagh	2011	165	6 (−.108)	No	–	CROSS	Mix	UN	62.0	–	DICHO	UN	UN	Mix
Metzger	2009	2483	1 (.056)	Yes	1.200	CROSS	OD	<16	49.0	91.0	DICHO	TEAM	UN	UN
Miller	2007	597	2 (.040)	Yes	2.777	LONG	SER	<16	45.0	30.2	Mix	UN	UN	Mix
Moesch	2009	1664	2 (−.050)	Yes	0.300	CROSS	OD	<16	47.8	–	INTEN	UN	UN	UN
Paetsch	1997	962	6 (.072)	Yes	1.659	CROSS	OD	UN	51.0	–	INTEN	UN	UN	UN
Raithel	2004	263	3 (.210)	Yes	–	CROSS	Mix	<16	49.0	–	DICHO	TEAM	UN	OUT
Reingle	2013	2165	2 (.045)	Yes	2.748	LONG	SER	<16	49.9	86.6	INTEN	UN	UN	UN
Roman	2013	390	2 (.034)	Yes	3.621	CROSS	OD	<16	46.6	96.5	INTEN	IND	UN	UN
Schafer	1969	585	1 (−.134)	Yes	1.782	CROSS	OD	UN	100	–	DICHO	UN	UN	SCH
Segrave	1982	1935	35 (−.191)	Yes	–	CROSS	Mix	Mix	Mix	Mix	DICHO	UN	UN	OUT
Segrave	1984	1693	3 (−.096)	Yes	0.730	CROSS	OD	UN	Mix	–	DICHO	TEAM	UN	SCH
Sokol-Katz	2006	4063	15 (−.035)	Yes	–	CROSS	OD	UN	100	–	DICHO	Mix	Mix	SCH
Thompson	1999	7733	4 (−.051)	No	–	CROSS	Mix	<16	49.2	4.0	INTEN	TEAM	UN	SCH
Tolk	2003	6504	25 (.063)	No	–	CROSS	OD	Mix	Mix	Mix	DICHO	UN	UN	SCH
Van der Laan	2009	598	1 (−.037)	No	–	CROSS	OD	UN	82.1	37.6	DICHO	UN	UN	OUT
Watkins	2000	582	70 (.055)	No	–	CROSS	Mix	UN	46.8	–	Mix	Mix	Mix	UN
Wilson	2010	314	5 (.084)	Yes	1.200	LONG	OD	UN	52.0	68.0	DICHO	Mix	Mix	OUT
Wong	2005	578	3 (−.051)	Yes	2.777	CROSS	Mix	UN	46.4	–	INTEN	UN	UN	UN
Yang	2000	818	1 (.170)	No	–	CROSS	OD	>16	50.2	–	INTEN	UN	UN	UN
Yin	1999	1326	8 (−.057)	Yes	.593	CROSS	Mix	<16	Mix	92.0	INTEN	UN	UN	SCH

*N* = number of participants; # *r* (*M*) = number of effect sizes (mean); impact factor = impact factor of journal; design = cross-sectional or longitudinal; outcome = type of offense; % male = percentage of males in sample; % minority = percentage non-Caucasian; team sports = team sports versus individual sports; contact sports = contact sports yes/no; setting = setting of sports participation; CROSS = cross-sectional design; LONG = longitudinal design; Mix = study contains different categories of moderator variables; OD = overall delinquency; PRO C = property crime; PRO D = property damage; PET = petty crimes; SER =  serious/violent crimes; UN = variable unspecified in study; TEAM = team sports; IND = individual sports; CON = contact sports; SCH = school setting; OUT = out of school setting

### Coding the Studies and Potential Moderators

The first author of this article coded the included studies according to the suggestions of Lipsey and Wilson (2001). The dependent variable in this meta-analysis was juvenile delinquency. The independent variable was sports participation. Ten studies (*#ES* = 46) were double coded by the first author and a research assistant. It is common to calculate the inter-rater agreement in a meta-analysis, because in addition to categorical variables, we also coded continuous variables. The inter-rater reliability proved to be good with 94 % agreement between the two coders.

The potential moderators of the association between sports participation and juvenile delinquency were grouped into offense, study, sample, and sports characteristics. The type of offense measured in the included studies was first coded as a string variable. After all studies were coded, we distinguished five types of offenses, based on the available data: overall delinquency, property crime (i.e., theft, shoplifting, stealing), property damage (i.e., vandalism), violent/serious crime (i.e., armed robbery, violent assault), and petty crime (i.e., minor offenses other than property crime or property damage).

The type of offense was coded as moderator variable, because different developmental trajectories towards different offense types have been showed (Moffitt 1993). Moreover, a commonly used argument supporting the association between sports participation and lower levels of engagement in delinquency is that athletes are just too busy to commit crimes (Hirschi 1969; Osgood et al. 1996; Schafer 1969). This seems specifically relevant when it comes to minor, opportunistic crimes (like petty crimes or property damage), because these crimes particularly originate from boredom and opportunity (Hirschi 1969; Osgood et al. 1996; Schafer 1969). Furthermore, it is possible that athletes withdraw from more serious crimes, as a possible sanction may jeopardize their opportunity to play (Miller et al. 2007). On the other hand, acting out may be part of the athletes’ culture, which can result in the engagement of minor delinquent behaviors, such as property damage and petty crimes (Miller et al. 2007). Therefore, the relationship between sports participation and juvenile delinquency may be moderated by offense type. In the majority of the studies (92 %) delinquency was measured by means of self-report. In four studies (8 %; *#ES* = 7) delinquency was measured through file information or official data. The effect of this possible moderator could not be assessed, because the numbers were too small to obtain sufficient statistical power.

We coded several study characteristics that may influence the strength of the relationship between sports participation and juvenile delinquency. First, the impact factor of the journal in which the study was published (continuous variable) was coded, because the impact factor is a first indication of study quality (Saha et al. 2003). Second, the year of publication (continuous variable) was coded, because we expected that the quality of older studies was lower than the quality of more recent studies, as the statistical and methodological knowledge has increased largely in social research over the last decades. Finally, the study design was coded (cross-sectional vs. longitudinal designs), as cross-sectional studies measure the relationship between sports participation and juvenile delinquency at one point in time, and longitudinal studies are able to take the developmental aspect of the relationship between sports participation and juvenile delinquency into account.

As sample characteristics we coded the proportion of males (continuous variable) and the proportion of youth with a minority background (non-Caucasian) in the sample (continuous variable). Gender is a potential moderator, because there are gender differences in developmental pathways towards delinquency and differences in benefits of leisure activity for boys and girls (Fredricks and Eccles 2006, 2008; Wong et al. 2010, 2013). Ethnicity was coded as a potential moderator, as it is unknown how well the findings of previous research generalize across ethnic groups (Fredricks and Eccles 2008).

Multiple sports characteristics were coded as potential moderators, because the type and setting of the sports activities might be significant in whether sports participation is positively, negatively or not related to juvenile delinquency. We coded whether the type of sports were team sports or individual sports. Team sports have been related to positive developmental outcomes because these sports promote the immediate practice of social skills (Ewing et al. 1996). On the other hand, Rutten et al. (2007) found that soccer players tend to show more antisocial behavior than swimmers. Whether sports were contact sports or non-contact sports was also coded as a potential moderator, because previous studies have found that young athletes in contact sports report more delinquent and violent behavior than athletes in non-contact sports (Levin et al. 1995; Endresen and Olweus 2005). Finally, it was coded whether the sports activities took place in a school or out-of-school setting. Sports in a school setting often involve skilled coaches, whereas the out-of-school setting often involves volunteers who do not necessarily have a pedagogical background or lack specific coaching skills (Ewing et al. 1996). Moreover, within the school setting there is often consultation between the school and the coach, which can contribute to a positive effect on the development of the participants (Perkins and Noam 2007).

### Calculation and Analysis

Effect sizes were transformed into correlation coefficient *r*. A positive correlation indicated that athletes are more delinquent than non-athletes, whereas a negative correlation can be interpreted as athletes being less delinquent than non-athletes. Effect sizes were calculated using the calculator of Wilson (2013) and formulas from Lipsey and Wilson (2001). If an article only mentioned that the relationship was not significant, an effect size was coded as zero (Lipsey and Wilson 2001), and a sensitivity analysis was conducted to test if this decision affected overall results. We also performed a sensitivity test to see if the inclusion of the adjusted effect sizes affected the overall results.

Continuous variables were centered on the mean, and categorical variables were recoded into dummy variables. Extreme values of the effect sizes (>3.29 *SD* from the mean; Tabachnik and Fidell 2013) were adjusted by winsorizing these outliers. Four outliers were identified at the lower bound of the distribution (range *r* = −.6790 to −.4170), they were winsorized to the value of *r* = −.4090. One outlier was identified at the upper bound of the distribution (*r* = .6690), this outlier was winsorized to the value of *r* = .4299. Correlation coefficients *r* were recoded into Fisher z-values (Lipsey and Wilson 2001). After the analyses, the Fisher z-values were transformed back into correlation coefficients for interpretation and reporting. Standard errors and sampling variance of the effect sizes were estimated using formulas by Lipsey and Wilson (2001).

By including multiple effect sizes per study, the assumption of independent effect sizes that underlie classical meta-analytic strategies was violated (Hox 2002; Lipsey and Wilson 2001). To deal with the interdependency of effect sizes, we applied a multilevel approach to the present meta-analysis as suggested by Van den Noortgate and Onghena (2003). A multilevel approach has the advantage that it accounts for the hierarchical structure of the data, where the effect sizes are nested within the studies. Therefore, all information in the studies can be preserved and maximum statistical power is generated, which allows comprehensive moderator analyses to assess the influence of offense, study, sample, and sports characteristics on the relationship between sports participation and juvenile delinquency (Van den Noortgate and Onghena 2003). We used a 3-level random effects model to account for three levels of variance, including the sampling variance for each effect size (level 1), the variance between effect sizes within a study (level 2), and the variance between the studies (level 3) (Wibbelink and Assink 2015). The meta-analysis was conducted in R (version 3.2.0) with the metafor-package, employing a multilevel random effects model (Houben et al. 2015; Van den Bussche et al. 2009; Viechtbauer 2010). This model is adequate and often used for multilevel meta-analyses, and in general superior to the fixed-effects approaches used in traditional meta-analyses (Van den Noortgate and Onghena 2003).

To estimate the model parameters the restricted maximum likelihood estimate (REML) was applied (Van den Noortgate and Onghena 2003). The Knapp and Hartung-method (2003) was performed to test individual regression coefficients of the models and for calculating the corresponding confidence intervals. The Knapp and Hartung-method (2003) has the advantage that it reduces Type I-errors (Wibbelink and Assink 2015). Likelihood ratio tests were used to compare the deviance scores of the full model and the models excluding the variance parameters of level 2 or 3, making it possible to determine whether significant variance is present at the two levels (Wibbelink and Assink 2015). In case there was significant variance on these two levels, the distribution of effect sizes was considered to be heterogeneous. This indicates that the effect sizes could not be treated as estimates of a common effect size, and moderator analyses were performed. For models including moderators, an omnibus test of the fixed-model parameters was conducted, which tests the null hypothesis that the group mean effect sizes are equal. Therefore, the test statistics of the moderator analyses were based on the F-distribution.

Although we made several efforts to prevent publication bias by our search strategy, this could not guarantee the absence of publication bias. In order to assess the influence of publication bias, we first tested funnel plot asymmetry according to Egger’s method (Egger et al. 1997). A funnel plot is a scatter plot of the effect sizes against the effect size’s precision (the inverse of the standard error). In case of publication bias, a gap in the effect size distribution would be present, showing an asymmetrical funnel plot and a significant Egger’s test. Second, we performed a trim and fill procedure (Duval and Tweedie 2000) by drawing a trim and fill plot in MIX 2.0 (Bax 2011). The trim and fill procedure corrects for funnel plot asymmetry by imputing estimated missing effect sizes that are calculated on the basis of existing effect sizes. If the trim and fill plot showed missing effect sizes, we imputed these estimated effect sizes of missing studies to the meta-analytic data, and reran the multilevel meta-analysis in R, as this shows the influence of the estimated missing data on the overall effect of the meta-analysis. Finally, the skewness of the effect size distribution was calculated in SPSS, because if publication bias is present, a skew distribution of the effect sizes would be expected (Begg and Mazumdar 1994).

## Results

Table 2 presents the results of the multilevel meta-analysis. The overall association between sports participation and juvenile delinquency can be found in this table, as well as the results of the moderator analysis. Only moderator variables with a significant contribution to a better fit of the model are reported in this table.

**Table 2:** The overall results and moderator effects relationship between sports participation and juvenile delinquency

	# study	# ES	*β*_0_ (mean *r*)	*t*_0_	*β*_1_	*t*_1_	*F*(d*f*_1_, d*f*_2_)
Overall association sports and juvenile delinquency	48	431	.005	0.323			
Overall association after trim and fill procedure	53	445	−.022	−1.219			
*Moderator variables*							
Type of offense	48	431					*F*(4,426) = 1.389
Overall delinquency (RC)			.009	0.604			
Property crime			−.011	−0.583	−0.020	−1.357	
Property damage			.016	0.799	0.007	0.398	
Serious/violent crime			−.012	−0.711	−0.022	−1.664	
Petty crime			.020	0.817	0.010	0.478	
Study characteristics							
Publication year (continuous)	48	431	−.000	−.008	0.002	1.928	*F*(1,429) = 3.719
Impact factor (continuous)	33	179	.005	0.382*	0.034	2.766**	*F*(1,177) = 7.650**
Type of study	48	431					*F*(1,429) = 6.387*
Cross-sectional (RC)	40	381	−.007	−0.494			
Longitudinal	8	50	.074	2.396*	0.081	2.527*	
Sample characteristics							
Proportion male (continuous)	46	416	.008	0.572	−0.030	−2.204*	*F*(2,414) = 4.856*
Gender (post hoc analysis)	46	416					*F*(2,413) = 4.259*
Male sample			−.013	−0.774			
Mixed sample			.013	0.868	0.026	1.859	
Female sample			.027	1.551	0.040	2.875**	
Proportion ethnic minority (continuous)	27	225	.003	0.183	0.022	0.838	*F*(1,223) = 0.703
Sports characteristics							
Type of sports	22	141					*F*(1,139) = 7.889**
Team sports (RC)	19	114	−.003	−0.109			
Individual sports	5	27	.057	2.011*	0.059	2.809**	
Type of sports	9	90					*F*(1,88) = 2.593
Contact sports (RC)			.031	1.705			
Non-contact sports			.009	0.488	−0.022	−1.610	
Setting	30	292					*F*(1,290) = 6.094*
School setting (RC)	15	160	−.047	−1.782			
Out-of-school setting	15	132	.042	1.606	0.083	2.469*	

# studies = number of independent studies; # ES = number of effect sizes; *t*_0_ = difference in mean *r* with zero; *t*_1_ = difference in mean *r* with reference category; mean *r* = mean effect size (*r*); *F*(d*f*_1_, d*f*_2_) = omnibus test; RC = reference category

* *p* < .05; ** *p* < .01

### Overall Relationship Sports Participation and Juvenile Delinquency

No significant association was found between sports participation and juvenile delinquency (*r* = .005; 95 % *CI* −.023 to .033; *p* > .05), suggesting that there is no significant overall relationship between athletic status and the level of delinquent behavior in adolescents.

Sensitivity analysis excluding the adjusted effect sizes (effect sizes controlled for background characteristics) had little effect on the overall association between sports participation and juvenile delinquency (*r* = −.001; 95 % *CI* −.039 to .037; *p* > .05). The sensitivity analysis excluding the studies where a reported null effect was coded as *r* = 0 did not affect the overall association between sports participation either (*r* = .006; 95 % *CI* −.023 to .034; *p* > .05; # studies = 47; # ES = 424).

**Fig. 1:**
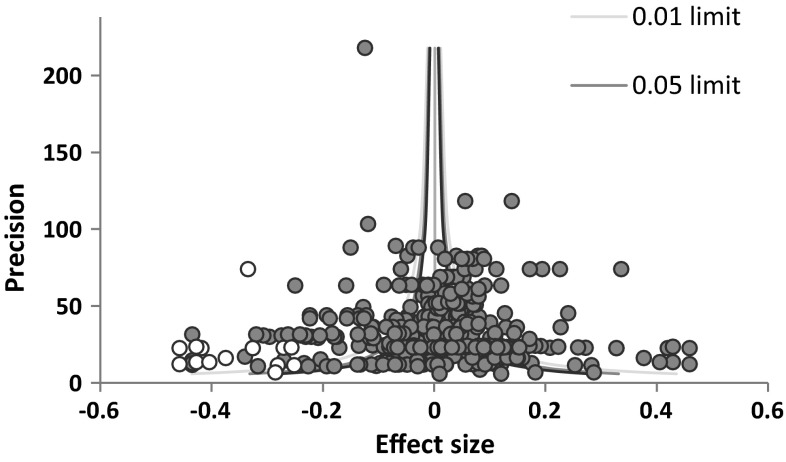
Trim-and-fill plot. *Note* graph from Bax (2011)

When checking for publication bias, first, Egger’s method did not indicate funnel plot asymmetry, because the intercept was not significant (*t* = −0.118, *p* = .906). However, the trim and fill plot revealed that there were some missing effect sizes, indicating publication bias. The trim and fill plot in Fig. 1 shows the imputation of estimated effect sizes with negative correlation coefficients (represented by the white dots) on the left side of the funnel. This indicates the absence of studies reporting that athletes are less delinquent than non-athletes. To check if this possible publication bias influenced the overall association between sports participation and juvenile delinquency, we added the imputed estimates to the data. Table 2 shows that imputation of the estimated effect sizes to the meta-analysis did not render results significantly (*r* = −.022, *p* > .05). Finally, the skewness test was not significant (*Z* = −1.263, *p* > .05), indicating that the effect size distribution was not skewed. Although there was some indication of publication bias according to the trim and fill analysis, we concluded that our findings are robust to the threat that excluded studies might have yielded a significant effect, because after imputation of the estimated effect sizes the overall mean effect size remained non-significant.

The likelihood ratio test comparing models with and without between-study variance (level 3) showed that significant variance was present at the between-study level ($\upsigma_{\text{level 3}}^{2} = \, 0.007$, χ^2^(1) = 215.784; *p* < .0001). The variance between the effect sizes within studies (level 2) was significant as well ($\upsigma_{\text{level 2}}^{2} = \, 0.005$, χ^2^(1) = 1965.307; *p* < .0001), indicating a heterogeneous effect size distribution. About 4 % of the total effect size variance was accounted for the sampling variance (level 1), 39 % for the variance between effect sizes within studies (level 2), and 57 % for the variance between studies (level 3). In case of heterogeneous effect size distributions, moderator analyses are advised to assess whether the variance between the effect sizes can be explained by certain factors, regardless of the significance of the overall effect size. Therefore, we conducted moderator analyses on offense, study, sample, and sports characteristics to examine the strength of the relationship between sports participation and juvenile delinquency. Table 2 shows the results of the moderator analyses.

### Type of Offense

The type of offense did not moderate the relationship between sports participation and juvenile delinquency (*F*(4,426) = 5.556; *p* > .05). The associations between sports participation and respectively property crime, property damage, serious/violent crime, and petty crime did not deviate from the association between sports participation and overall delinquency. None of the specific types of offenses were significantly related with sports participation.

### Study Characteristics

Several study characteristics had a moderating effect on the relationship between sports participation and juvenile delinquency (see Table 1). The impact factor of the journal in which the study was published significantly moderated the relationship between sports participation and juvenile delinquency (*F*(1,177) = 7.650; *p* < .01). Among published articles, stronger, positive associations between sports participation and juvenile delinquency were found for studies in the more frequently cited journals. Moreover, the type of study seemed to influence the relationship between sports and juvenile delinquency (*F*(1,429) = 6.387; *p* < .05). Only among studies using longitudinal designs significant results were found (*r* = .074), indicating that athletes were more delinquent than non-athletes. Furthermore, the year of publication did not moderate the strength of the relationship between sports participation and juvenile delinquency.

### Sample Characteristics

Only gender moderated the relationship between sports and juvenile delinquency (*F*(2,413) = 4.856; *p* < .05). Studies with lower proportions of males in the sample, showed more positive correlations with juvenile delinquency. To be able to interpret this result more clearly, we conducted post hoc analysis with a more stringent α-level of .025, with all-male, mixed, and all-female samples in the analysis. In this post hoc analysis, gender significantly moderated the relationship between sports participation and delinquency (*F*(2,413) = 4.259; *p* < .025). The correlations between sports participation and juvenile delinquency significantly differed in all-female samples from the all-male samples. However, the individual categories did not show significant correlations between sports participation and juvenile delinquency (male samples *r* = −.013, mixed samples *r* = .013, female samples *r* = .027; *p* > .05). The proportion of adolescents from ethnic minority groups did not moderate the relationship between sports participation and juvenile delinquency.

### Sports Characteristics

Moderating effects were found for multiple sports characteristics. The type of sport had a moderating effect on the relationship between sports participation and juvenile delinquency (*F*(1,139) = 7.889; *p* < .01). Individual sports showed a significant mean association (*r* = .057), indicating that athletes of individual sports were more delinquent than non-athletes, whereas no relationship between sports participation and juvenile delinquency was found in team sports. Further, the setting of the sports participation (whether the sports were school-based or in an out-of-school setting) moderated the relationship between sports and juvenile delinquency (*F*(1,290) = 6.094; *p* < .05). However, the individual categories did not show significant correlations for the relationship between sports participation and juvenile delinquency (school setting mean *r* = −.047, out of school setting mean *r* = .042, both *p* > .05). Finally, whether or not the athletes participated in contact sports did not moderate the relationship between sports participation and juvenile delinquency.

## Discussion

Sports participation plays an important role in the lives of adolescents. Much is known about the positive associations between sports participation and psychosocial health (Eime et al. 2013; Janssen and LeBlanc 2010), but theoretical and empirical knowledge about the relationship between sports participation and juvenile delinquency is lacking (Coakley 2002; Farb and Matjasko 2012; Nichols 2007). Nevertheless, sports are used worldwide to prevent juvenile delinquency (Cameron and MacDougall 2000; Hartmann 2003; Kelly 2013; Miller et al. 2007; Nichols 2007; Sandford et al. 2006). This multilevel meta-analysis is the first systematic review that examined the association between sports participation and juvenile delinquency by synthesizing previous research on sports participation and juvenile delinquency.

Overall, no significant association was found, indicating that there was no significant relationship between sports participation and juvenile delinquency (*r* = .005). This result was maintained even after controlling for possible publication bias by a trim and fill procedure. However, the distribution of effect sizes was heterogeneous, indicating that there was variation between the effect sizes within and across studies, possibly explained by moderators. Therefore, we conducted moderator analyses on offense, study, sample, and sports characteristics.

Moderator analyses showed that the type of offense did not influence the relationship between sports participation and juvenile delinquency, and that sports participation was not associated with overall delinquency, serious/violent crime, property crime, property damage, or petty crime. Some study, sample, and sports characteristics did influence the relationship between sports participation and juvenile delinquency. Athletes were more delinquent than non-athletes in studies published in more frequently cited journals and using longitudinal designs. Furthermore, gender influenced the relationship between sports participation and juvenile delinquency. In all-female samples, more positive correlations were found than in all-male samples. Finally, the setting of the sports environment and whether it was a team or individual sport moderated the relationship with juvenile delinquency. Athletes participating in an out-of-school setting appear to have less favorable outcomes regarding juvenile delinquency compared to athletes in a school setting. Individual sports were associated with less delinquency, whereas for team sports no significant results were found. However, it has to be noted that, although there were significant moderating effects from study, sample, and sports characteristics, the correlations found in the moderator analyses were extremely small (in all cases *r* < .08), and it is expected that the practical or clinical value of these findings is minimal.

From the results of the current meta-analysis, we conclude that, in general, sports involvement is not reliably related to more or less juvenile delinquency, and that this non-significant association is only marginally affected by the moderating factors that were assessed in the current study. This conclusion has some important theoretical implications. Contrary to many criminological theories, such as Hirschi’s (1969) theory of social bonds, the boredom theory (Schafer 1969), and the routine activities theory (Cohen and Felson 1979), sports alone fail to protect youth from delinquent behaviors. In line with other researchers and theorists, we conclude that sports participation by itself may not be enough to increase protective social bonds and to eliminate boredom and opportunities for crimes in order to reduce delinquent behavior (Agnew and Petersen 1989; Tappan 1949; Wong 2005). On the other hand, contrary to theories assuming that sports participation is associated with more delinquency (i.e., the theories on the antisocial influence of sports because of the competitive element of sports and the alcohol consumption culture), sports do not seem to increase delinquent behavior among youth either. One explanation of the finding of no significant overall effect could be that sports participation is not associated with juvenile delinquency at all. The assumed positive influences of sports may not be strong enough to affect behaviors and skills outside the sports context, and to protect against juvenile delinquency (Shields and Bredemeier 1995; Tappan 1949). Another explanation we would like to propose is the possibility that protective influences of sports participation may be attenuated by the negative influences of sports participation on the development of juvenile delinquency. In this view, we acknowledge the potential positive influences of sports, but also consider a possible risk of sports participation regarding the development of juvenile delinquency.

Our suggestion that the positive and negative influences of sports participation on juvenile delinquency may countervail each other has implication for the realization of an appropriate sports context. In the sports environment, the protective influences of sports on juvenile delinquency must be highlighted, and the negative influences on the development of juvenile delinquency confined. The results of the current meta-analysis showed that more favorable outcomes (i.e., less delinquency) were found in sports participation within school settings and in team sports. This may be explained by the involvement of skilled coaches in school settings, while the out-of-school setting often involves volunteers who do not necessarily have a pedagogical background or lack specific coaching skills (Ewing et al. 1996). Further, within the school setting, there is often consultation between the school and the coach, which can contribute to a positive effect on the development of the participants (Perkins and Noam 2007). Team sports may have been related to less delinquency, because these sports promote the immediate practice of social skills (Ewing et al. 1996).

Previous studies have offered some implications for the development of an adequate sports context as well. The beneficial effects of sports can be expected when there is a climate of “fair play”-mentality and when team play, the development of athletes, and acquiring skills are considered more important than performance (Guivernau and Duda 2002; Miller et al. 2005; Rutten et al. 2007). The sports coach plays a significant role in providing an adequate sports context that leads to positive psychological outcomes in athletes (Côté and Gilbert 2009; Ntoumanis et al. 2012; Smith et al. 2007). Knowledge of education, interpersonal skills, the ability to reflect upon oneself, and understanding of the developmental needs of individual adolescent athletes are important characteristics of coaches, which might positively affect the development of young athletes (Côté and Gilbert 2009). In sum, we argue that sports participation may protect against juvenile delinquency when the sports environment consists of elements that guarantee a positive and safe sports environment (Côté and Gilbert 2009; Rutten et al. 2007).

In the current meta-analysis, it was difficult to test our hypothesis of a protective influence of sports on juvenile delinquency when the sports environment is able to guarantee an appropriate context for development and negative aspects of sports are minimized. None of the included studies provided information about relevant characteristics of the sports environment, such as the quality of the relationship with the coach, the education of the coach, and the quality of the moral atmosphere of the sports environment (Rutten et al. 2007). Future research with longitudinal designs should focus on these contextual factors to understand more about the relationship between sports participation and juvenile delinquency, and mechanisms that contribute to positive developmental outcomes in adolescents.

There are some limitations of this study that need to be addressed. First, this study included non-published, non-peer reviewed manuscripts with weak study designs. Second, we combined unadjusted and adjusted effect sizes in the meta-analysis. This may be problematic, because the adjusted effect size may be smaller or larger than the related unadjusted effect size, which can affect the overall effect size (Aloe and Thompson 2013). On the other hand, the sensitivity analysis showed that the exclusion of the adjusted effect sizes had little effect on the overall relationship between sports participation and juvenile delinquency. Therefore, we argue it is justified to include the adjusted effect sizes in the meta-analysis, and with that, to prevent publication bias. Third, the included studies did not always provide detailed information about sample and sports characteristics. In the majority of studies, the independent variable was described as the general term “sports”. Previous research, as well as the current study, showed that specific characteristics of sports or the sports environment influence the relationship between sports participation and juvenile delinquency (Endresen and Olweus 2005; Rutten et al. 2007). However, because of the lack of a distinction between the different types of sports in the studies and characteristics of the sports context, we could only include a limited number of moderators. Finally, youth with more proneness towards delinquency may show lesser or greater chances to get involved in sports participation. Thus, the results of this meta-analysis may be influenced by self-selection bias (Fredricks and Eccles 2006). As the present meta-analysis consists of mostly cross-sectional studies aimed to assess the relationship between sports participation and juvenile delinquency, we refrain from making a causal statement about the effects of sports participation on juvenile delinquency.

Despite the limitations, the current meta-analysis has several strengths. First of all, this is the first systematic review on the relationship between sports participation and juvenile delinquency filling gaps in theoretical and empirical knowledge on two important topics in adolescence. Second, by using an advanced multilevel approach that allowed for the inclusion of multiple effect sizes per study, comprehensive moderator analyses were possible, leading to a better understanding of (the lack of) moderating influences. Third, we increased the comparability of the studies included in the meta-analysis by using a narrow definition of juvenile delinquency. Finally, we have made efforts to prevent publication bias by conducting an extensive systematic literature search and including unpublished studies. The advantage of including unpublished studies is that it increases the representativeness of the selected studies and decreases the chances of publication bias (Duval and Tweedie 2000). Moreover, we controlled for the possible publication bias by performing a trim and fill procedure. All in all, the strengths of this meta-analysis assure the representativeness of the finding of no overall significant relationship between sports participation and juvenile delinquency, providing an important contribution to the research on adolescence.

## Conclusions

Despite the large role of sports in the development of adolescence, little is known about the relationship between sports participation and juvenile delinquency. There is much controversy on whether sports participation should be perceived as a protective or a risk factor for the development of juvenile delinquency. This study aimed to provide more insight in the association between sports participation and juvenile delinquency. The findings of this multilevel meta-analytic review showed that, overall, sports participation was not related to juvenile delinquency. Some significant moderators were identified, but the influences of the study, sample, and sports characteristics examined in this review were minimal. We have explained these results by the suggestion that the alleged positive influences of sports may be countervailed by the supposed negative influences of sports. This has implications for the way that sports activities are implemented for adolescents. The sports context may amplify the positive elements of sports, such as the opportunity to form prosocial relationships with peers and the coach (Fredricks and Eccles 2005; Rutten et al. 2007), practice social skills (Vidoni and Ward 2009), and decrease the elements that may contribute to juvenile delinquency, such as the emphasis on competition (Stanger et al. 2013). Improving the pedagogical quality of the sports environment and including those measures in research on sports participation and psychosocial development may provide important knowledge to realize the potential positive influence of sports activities on juvenile delinquency.
